# Increasing dental zirconia micro-retentive aspect through ultra-short pulsed laser microstructuring: study on flexural strength and crystal phase characterization

**DOI:** 10.1007/s00784-021-04077-2

**Published:** 2021-08-17

**Authors:** Stephanie Assimakopoulos Garófalo, Martin Wehner, Andreas Dohrn, Marin Dean Bilandžić, Christian Roos, Richard Johannes Wierichs, Hendrik Meyer-Lueckel, Ana Cecilia Corrêa Aranha, Marcella Esteves-Oliveira

**Affiliations:** 1 Department of Restorative Dentistry, School of Dentistry, University of São Paulo, São Paulo, Brazil; 2 Fraunhofer Institute for Laser Technology (ILT), RWTH Aachen University, Aachen, Germany; 3 Institute of Mineral Engineering (GHI), RWTH Aachen University, Aachen, Germany; 4 Department of Restorative, Preventive and Pediatric Dentistry, Zmk Bern, University of Bern, Bern, Switzerland; 5 Special Laboratory of Lasers (LELO), Department of Restorative Dentistry, School of Dentistry, University of São Paulo, São Paulo, Brazil; 6 Department of Cariology, Endodontology and Periodontology, University of Leipzig, Leipzig, Germany

**Keywords:** Yttria-stabilized tetragonal zirconia, Lasers, Dental materials, Zirconia conditioning, XRD, Texturing, Bending

## Abstract

**Objectives:**

Although ultra-short pulsed laser (USPL) microstructuring has previously improved zirconia bond-strength, it is yet unclear how different laser-machined surface microstructures and patterns may influence the material’s mechanical properties. Therefore, the aim of this study was to assess the flexural strength of zirconia after different USPL settings creating three different geometrical patterns with structures in micrometer scale.

**Methods:**

One hundred sixty zirconia bars (3Y-TZP, 21 × 4 × 2.1 mm) were prepared and randomly divided into five groups (*n* = 32): no surface treatment (negative control-NC); sandblasting with Al_2_O_3_ (SB); and three laser groups irradiated with USPL (Nd:YVO_4_/1064 nm/2-34 J/cm^2^/12 ps): crossed-lines (LC), random-hatching (LR), and parallel-waves (LW). Bars were subjected to a four-point flexural test (1 mm/min) and crystal phase content changes were identified by X-ray diffraction. Surface roughness and topography were analyzed through 3D-laser-profilometry and SEM. Data were analyzed with parametric tests for roughness and Weibull for flexural strength (*α* = 5%).

**Results:**

LR (Mean[95%CI]: 852.0 MPa, [809.2–894.7]) was the only group that did not show a significantly different flexural strength than NC (819.8 MPa, [796.6–842.9]), (*p* > 0.05). All laser groups exhibited higher Weibull moduli than NC and SB, indicating higher reliability and homogeneity of the strength data. An increase of monoclinic phase peak was only observed for SB.

**Conclusion:**

In conclusion, USPL created predictable, homogeneous, highly reproducible, and accurate surface microstructures on zirconia ceramic. The laser-settings of random-hatching (12 ps pulses) increased 3Y-TZP average surface roughness similarly to SB, while not causing deleterious crystal phase transformation or loss of flexural strength of the material. Furthermore, it has increased the Weibull modulus and consequently material’s reliability.

**Clinical significance:**

Picosecond laser microstructuring (LR conditions) of 3Y-TZP ceramic does not decrease its flexural strength, while increasing materials realiability and creating highly reproducible and accurate microstructures. These features may be of interest both for improving clinical survival of zirconia restorations as well as enhancing longevity of zirconia implants.

## Introduction

Zirconium dioxide (zirconia) ceramic presents the highest strength among all dental ceramic materials [1, 2]. It has 3 principal phases: monoclinic (m) at room temperature, tetragonal (t) above ~ 1,170 °C, and cubic (c) above ~ 2,370 °C [3]. Enhanced strength and fracture toughness are obtained with the tetragonal phase, which is for pure zirconia not observed at room temperature. Therefore, currently, dental zirconia most commonly consists of 3 mol% yttria-stabilized tetragonal zirconia polycrystalline (3Y-TZP), which includes yttria (Y_2_O_3_) as dopant to stabilize the tetragonal phase at room temperature [2, 4]. This material has exceptional mechanical properties (i.e., high fracture toughness and high strength), due to its ability to undergo stress-induced crystal phase transformation from tetragonal to monoclinic (t-m transformation) [5, 6]. The t-m transformation is accompanied by a 3–5% volume increase, causing compressive stress that inhibits crack propagation, the so-called transformation toughening discovered in the late 1970s [7].

The final properties of zirconia will heavily depend on both the production processing steps during fabrication as well as on the introduction of surface damage after processing as a result from grinding, sandblasting or wear [8, 9]. As 3Y-TZP cannot be acid etched roughening the internal surfaces (as for example by means of sandblasting) before cementation has been recommended to improve adhesion to resin cements [10, 11]. However, both grinding with coarse diamond or carbide tungsten burs as well as sandblasting of the interior surfaces of 3Y-TZP ceramic restorations have been shown to result in contrary effects on material strength [12]. Although both generate surface irregularities and an increase in surface roughness, necessary for adhesive cementation, grinding typically result also in deep surface defects and reverse transformation toughening due to the high temperatures involved [13, 14]. Conversely, sandblasting has been shown to cause less deep surface flaws (ring cracks) and additionally increases the stress-induced tetragonal to monoclinic transformation (higher amount of monoclinic phase at the surface zone) resulting in an increase of protective surface compressive stress, which inhibits crack propagation and significantly increases flexural strength [13, 15]. Although controversial effects of sandblasting on flexural strength of dental zirconia ceramic have been reported, a recent systematic review and meta-analysis confirmed that sandblasting significantly improved the flexural strength of 3Y-TZP ceramic [16].

Overtime though, the initially increased flexural strength decreases at higher rate by sandblasted than for non-sandblasted materials [17, 18]. As a result, after aging of 3Y-TZP, there is no significantly difference in flexural strength of sandblasted and not-sandblasted materials. Furthermore, some researchers have advocated that sandblasted 3Y-TZP might be more susceptible to low temperature degradation [1, 19]. However, this is still controversial and other experiments have shown that only Y-TYP/Alumina zirconia and not 3Y-TZP was more susceptible to aging after sandblasting [20]. There is also evidence showing that an excessive increase of m-phase content (> 12–25%) [21] during transformation toughening is directly related with the degradation of the zirconia material [15, 19, 22]. Thus, it is yet unknown if there are better ways of machining zirconia to increase its surface roughness and adhesion, without increasing the m-phase content of the zirconia material.

Lately, ultra-short pulsed lasers (USPL) have been recognized as a promising tool for both machining as well as microtexturing 3Y-TZP in its final sintered (hard) state [23, 24]. The first process refers to machining dental restorations out of CAD-CAM blocks as well as dental implants out of 3Y-TZP cylinders [24, 25] and the second refers to creating micro- or nano-scaled patterns at the inner surface of restorations for improving boding [26, 27] or to patterning the outer surface of dental implants to improve osteointegration [28, 29]. All these processes involve laser-mediated ablation of zirconia at a minor or major extent. Indeed, efficient material removal can be achieved by laser processing 3Y-TZP with pulse durations from some tens of nanoseconds (ns) to femtoseconds (fs), which results in material removal rates ranging from 1.3 to 2.1 mm^3^/min [25, 30]. Even if quite similar material removal rates are obtained with longer nanoseconds pulses, laser microstructuring (texturing) with shorter pulses (in the ultrashort pulse range, i.e., tens to hundreds of pico- or femtoseconds) results in better zirconia surface quality, without microcracks [30–32] and also increases hydrophilicity [33]. Whereas longer pulses (i.e., tens of ns) clearly results in molten material at the surface, as it is a thermal-based process involving high surface temperatures [34, 35].

Material processing with ultrashort laser pulses occurs rather as a quasi non-thermal process. Ultrashort laser pulses interact with matter through non-linear effects at extremely high power densities exceeding the threshold for laser-induced optical breakdown (over 10^7^ W/cm^2^ and up to 10^15^ W/cm^2^) [36]. As the duration of a femtosecond laser pulse is so extremely short (1 billionth of a second), the heat generated cannot diffuse into the material [37]. This kind of laser-matter interaction is extremely precise, wavelength-independent and occurs mainly through photodisruption and plasma-induced ablation, also called “cold-ablation.”

Although there is clear evidence that pico- and femtosecond (ps and fs) laser texturing may improve both bonding [26, 27] to zirconia restorations as well as osseointegration to zirconia dental implants [28, 29], there are only few studies investigating the influence of this kind of laser-based surface microstructuring (or microtexturing) on one of the most important zirconia properties, namely the flexural strength. As in the case of laser applications, changing the irradiation conditions can cause totally different effects on the material being irradiated; it is of great importance to evaluate the influence of different conditions on the changes in mechanical properties of the zirconia ceramic. For example, longer pulse durations (10–60 ns) have been shown to cause in three studies a significant decrease in 3Y-TZP’s flexural strength [30, 35, 38]. On the other hand, pulses in the range of pico- and femtoseconds have shown to maintain the flexural strength of zirconia ceramic at the same level as that of non-irradiated (as-sintered) material [30, 32]. Thus, there is a clear tendency for better surface quality and maintenance of a high flexural strength with ps and fs laser microstructuring. However, not only the pulse duration but also the kind and geometry of surface patterns created on the dental zirconia may influence flexural strength. To the best of our knowledge, specially the direct influence of different surface patterns on flexural strength of 3Y-TZP ceramic has not been studied before. A detailed comparison between the studies of laser microtexturing of zirconia is also extremely difficult because in each study a different surface pattern has been investigated. As for example, solely parallel lines [35, 38], lines and pores [24], helical grooves [25] and in some other studies not much details are given about the laser-created surface patterns.

Therefore, the aim of the present study was to evaluate the surface roughness, surface morphology, and mechanical behavior of a zirconia ceramic after different combinations of USPL irradiation parameters and surface patterns. The null hypothesis tested was as follows: there is no significant difference in flexural strength and roughness means between 3Y-TZP ceramic receiving no treatment, sandblasting with Al_2_O_3_ or automated USPL irradiation creating different surface patterns. Additionally, crystal phase changes occurring after sandblasting and laser irradiation were analyzed, as possible phase changes also have a considerable influence on 3Y-TZP mechanical properties.

## Materials and methods

### Ceramic samples

A 3 mol% Y-TZP (3Y-TZP) partially sintered ceramic (VITA-In-Ceram®-YZ, Model: YZ-65/40 s, Lot: #15,040, Vita-Zahnfabrik, Bad Säckingen Germany), normally produced for a later CAD/CAM milling process and containing approximately 94 mass% of tetragonal zirconium dioxide, was used in this study. Out of this material, 160 bar-shaped samples of 24 × 4 × 2.1 mm were manufactured according to the ISO-6872 (2015) [39]. Shortly, the initial pre-sintered blocks (65 × 40 × 16 mm) were cut using a water-cooled diamond band at slow speed (Diamond band saw 300, Exakt, Norderstedt, Germany) into 29 × 5x2.6 mm bars. A 45°-chamfer was prepared at major edges and all dimensions were measured with a digital caliper (Mitutoyo Corporation, Japan, accuracy: ± 0.001 mm). The 45° chamfer was made by fixing each sample in a microscope slide with wax and leaning them at a 45 degree angle over the silicon carbide paper. The tolerance for the sample dimensions was set at 0.2 mm in width/ length and 0.1 mm in height. The samples were measured in three areas (right, middle, and left), regarding their height, to assure plane parallel test pieces. Afterwards, samples were ground with wet silicon carbide papers (Grits: 600/1200/2400), sintered according to manufacturer’s instructions (1530 °C/ 2 h/ Programat S1 oven; Ivoclar Vivadent AG). After sintering, bar-shaped samples with the final dimensions (24 × 4 × 2.1 mm) were randomly assigned to five groups (*n* = 32) receiving different surface treatments. Zirconia grains of an average size of 0.72 μm (± 0.08 μm) are usually obtained under this sintering protocol [40].

### Determination of ablation threshold and ablation efficiency

In order to determine the ablation threshold for the laser irradiation of the 3Y-TZP ceramic, laser irradiation was performed at 1064 nm wavelength (Neodymium-doped yttrium orthovanadate—Nd: YVO_4_, Trumicro-5050, Trumpf, Ditzingen, Germany) with fluences of 2 to 34 J/cm^2^ and average power of 1 to 11 W, in four different pulse rates: 50 kHz, 100 kHz, 400 kHz, 800 kHz. After the surface treatments, average surface roughness (Ra), maximum “peak” to “valley” distance (Rz) as well as average groove depth (h) were determined using a 3D laser scanning microscope (profilometer, VK-X110, Keyence, Osaka, Japan). Additionally, for the analysis of surface morphology, a scanning electron microscope (SEM, Leo-1455VP, Zeiss, Oberkochen, Germany) was used and images were obtained at 20 kV, under 5–10 mbar pressure and working distance of 13–14 mm (100–4000 × magnifications). Before examination, samples were rinsed with 96% ethanol, gently air-dried, mounted on metallic stubs, gold-sputtered.

### Study design

**Fig. 1:**
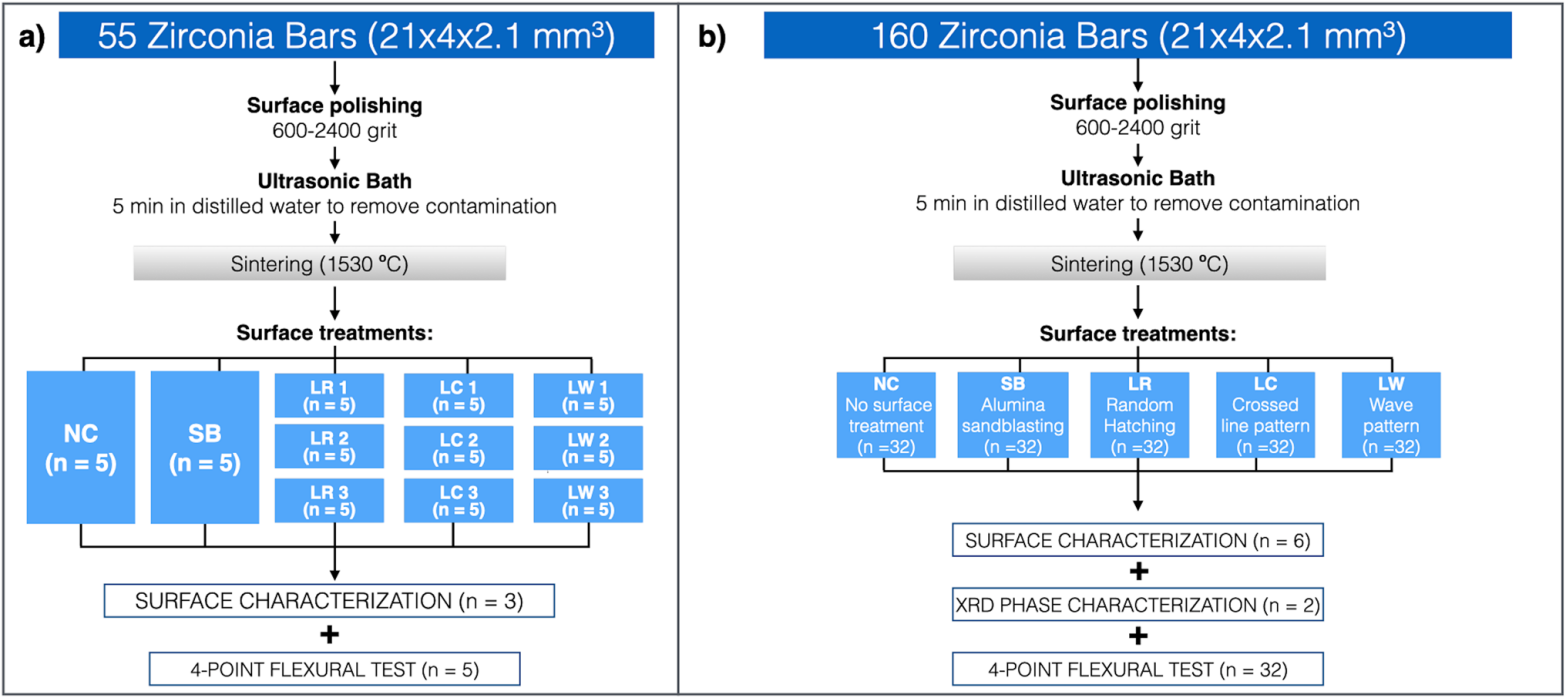
Flow chart of the pilot (a) and the main study (b). An overview of number of dental zirconia bars, types of surface treatments and description of the analyses performed (flexural strength, surface analysis and XRD phase characterization) in both studies is shown

**Fig. 2:**
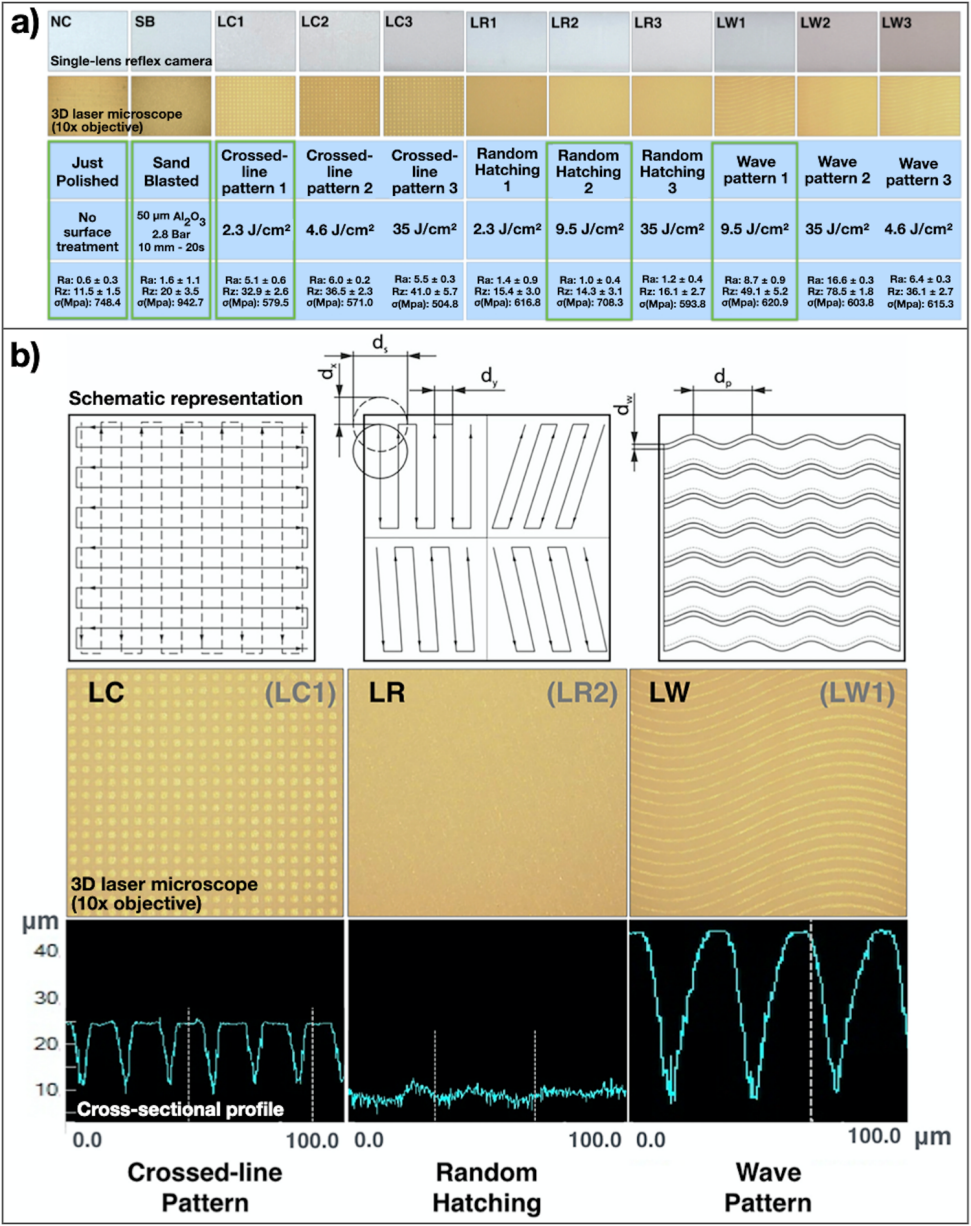
**a)** Images of the pilot study (definition of irradiation parameters and patterns). Bottom part (blue): treatments (parameters and patterns); surface roughness analysis (Ra and Rz) and mean flexural strength The parameters causing the highest flexural strength and the least color change are marked in green and were chosen for the study (LC1, LR2 and LW1). **b)** Laser scanning strategies for the three surface patterns chosen in the pilot study (LC: crossed-lines, LR: random hatching and LW: wave pattern). Upper row: Schematic drawing of the laser scanning paths programed to obtain the different surface patterns (dx = beam offset in scribing direction; dx = v/f; dy = distance between scanlines; ds = laser beam diameter; dw = distance between wave pattern; dp = distance between periods). For LR the 4 different paths described in the figure were randomly changed by an algorithm in the software controlling the laser microstructuring. Middle row: representative laser microscopy (10 × enlargement) of laser irradiated zirconia. Lower row: cross-sectional profile of each irradiation pattern. The shallowest modifications can be seen in group LR, while the deepest grooves in LW, which are about twice as deep as the grooves observed in LC

This study was divided into two parts. First, a pilot study with the main purpose of screening for the appropriate laser irradiation conditions and patterns. Subsequently, a main study, where the most appropriate irradiation settings (from the pilot study), were tested for their influence on zirconia’s four-point flexural strength (using a higher number of samples, *n* = 32) and on the relative monoclinic content (Fig. 1). According to the international standards for flexural strength testing of dental ceramics (ISO-6872) [39], thirty samples per group (*n* = 30) are recommended for the four-point flexural strength test. To account for possible losses during sample preparation, two samples more per group were included. For both studies, the surface treatments were applied over the whole surface of the test bars (24 × 4 mm^2^ area) and were conducted as follows:

- No surface treatment as negative control (NC);
- Sandblasting (SB), as positive control, with 50 µm aluminum oxide particles (KoroxTM, BEGO, Qébec, Canada) for 20 s (2.8 bar, 10 mm distance, Sandblaster: Basic Quattro, Renfert, Hilzingen, Germany) [20];
- Irradiation with an ultra-short pulsed laser with crossed-lines pattern (LC);
- Irradiation with an ultra-short pulsed laser with random hatching pattern (LR);
- Irradiation with an ultra-short pulsed laser with wave pattern (LW);

In the pilot study, for each surface pattern, three different energy densities were tested (LC1, LC2, LC3, LR1, LR2, LR3, LW1, LW2, LW3) (Fig. 2a).

### Screening of new laser irradiation settings and patterns (pilot study)

Based on the knowledge that the sharp internal angles of the restorative materials or dental cavities may lead to stress concentration [8, 9] and knowing that the laser crossed-line pattern creates relative sharp internal angles within the first micrometers of the zirconia blocks, we have speculated that a previously established irradiation condition [27] could facilitate crack formation, thus not being the most ideal for the flexural strength of the treated zirconia. Since this previous study was, to our best knowledge, the first investigating this kind USPL-machined pattern to improve adhesion to zirconia, and its impact on the mechanical properties was unclear, care was taken to conduct a pilot study, searching for additional laser irradiation settings and surface patterns. Specially the creation of surface microstructures and groves, without sharp angles (such as random forms and waves), which could not only increase surface area and microretention (favouring adhesion to resin cements), but also reduce the chances of weakening the zirconia were searched. As no references were available as regards best surface patterns, best energy densities, pulse durations, scanning speed, and so on, a large screening of laser parameters was conducted in order to delimitate the huge amount of possible variable laser settings and scanning patterns for this application. After this general screening using a stereo microscope, the 9 most adequate set of parameters were chosen for more detailed surface characterization and for a flexural strength test (Fig. 1a) “The parameters causing the least visible surface damage (stereo microscopy analysis), having higher roughness than the control group and resulting in the highest flexural strength means (*n* = 5) were included in the main study (Fig. 2a and b).” In LC and LW scanning patterns, the surface is partially not affected, meaning that in the space between the clearly separated lines, the original untreated surface remains. As opposite to that by the random hatching scanning strategy each point of the surface was covered by a laser pulse, which resulted in the removal of a complete surface layer and a surface structure reflecting the orientation and number of different hatching directions.

### Laser irradiation

After screening for the appropriate laser irradiation parameters and patterns, irradiation settings were chosen (Table 1). Surface treatments tested were as follows: no surface treatment as negative control (NC); sandblasting (SB) with 50 µm aluminum oxide particles for 20 s (2.8 bar, 10 mm distance) [11, 15]; and USPL irradiations with an automated processing system, using 12 ps pulses, high scanning speed and generating the following surface scanning patterns (Fig. 2b): crossed-lines (LC); random hatching (LR) and parallel waves (LW).

**Table 1:** Description of the ultra-short pulsed laser parameters. All having pulses of only 12 trillionths of a second (10^–12^ s) pulse duration

Description	Laser Random Hatching Pattern (LR)	Laser Crossed-Lines Pattern (LC)	Laser Wave Pattern (LW)
Crystal	Nd: YVO_4_	Nd: YVO_4_	Nd: YVO_4_
Wavelength	1064 nm	1064 nm	1064 nm
Output Power	5 W	9 W	5 W
Energy per Pulse	50 µJ	11 µJ	50 µJ
Average Fluence	9.5 J/cm^2^	2.3 J/cm^2^	9.5 J/cm^2^
Pulse Rate	100 kHz	800 kHz	100 kHz
Pulse Duration	12 ps	12 ps^*^	12 ps
Distance of Irradiation	100 mm	100 mm	100 mm
Scan speed	1 m/s	3 m/s	1 m/s
Number of Scan Repetitions	10	12	20
Irradiation Pattern	Random Hatching	Crossed-lines Pattern	Wave Pattern
Beam diameter	25 µm	25 µm	25 µm
Distance Between Lines (‘dy’: for LC and LR, ‘dw’: for LW)	10 µm	35 μm	4 µm
Average Ablation Depth	≈30 μm	≈15 μm	≈32 μm

^*^ps picoseconds

A picosecond laser (Trumicro-5050, Trumpf, Ditzingen, Germany) fitted to a 3D-machining-center (Lasertec-50-Shape, Sauer, Pfronten, Germany) was used. The laser scanner irradiated an area of 24 × 4 mm^2^ having the different irradiation patterns described above (LC, LR, LW). The distance between subsequent pulses was about 4 µm, considering both the pulse and the feed rate [41]. Laser beam focus was adjusted individually for each block. Total ablation depth was 15–35 µm (Table 1). One complete surface scan was considered one repetition, as previously described [42]. No water cooling was used and the irradiation time was about 5 s/repetition.

For the surface pattern LR, the randomization of the scan directions was performed by the correspondent software “Laser Programming System for Windows” (Lasertech-50 Precision Tool, DMG Mori, Tokyo, Japan). Hereby the direction of the scan lines was changed by build-in algorithm in a random manner between each layer. Usually after 10 layers, textures were not visible anymore on the specimen’s surface.

The laser parameters presented in Table 1 were selected using our experience from previous studies both as regard laser-zirconia [27] as well laser-enamel interactions [42, 43], in which we could observe that specially low-fluences below 3–10 J/cm^2^ combined to very low pulse durations tend to avoid surface damage and are therefore specially interesting for dental applications.

### Determination of ablation threshold and ablation efficiency

The average ablation rate was calculated using the groove depth (measured with Keyence VX6000 video microscope) and the total pulse number per site. The calculation of total number of pulses per site was done using the following equation:

$${N}={D_F}/{d_x}*{D_F}/{d_y}*n$$

where *D*_*F*_ represents the beam diameter, *d*_*x*_ the beam offset in scribing direction (*d*_*x*_ = *v / f)*, *d*_*y*_ the distance between scan lines; *v* the laser beam feed rate; *f* the laser pulse repletion rate, *n* the number of scan layers. Subsequently, the ablation rate (*h*) in µm/pulse was calculated as follows:

$$h=t/{N}$$

where *t* represents the total ablation depth.

### Scanning electron microscopy (SEM)

Change in the microstructure of the ceramic material was investigated through SEM (*n* = 6). Samples were rinsed with 96% ethanol, gently air-dried, mounted on metallic stubs, gold-sputtered (Evaporation-unit) and subsequently examined under a SEM microscope (Leo-1455VP, Zeiss, Oberkochen, Germany) at 20 kV (100–4000 × magnifications)), under 5 to 10 mbar, and working distance of 13 to 14 mm.

### Surface roughness

Topography changes in the treated surfaces were analyzed (*n* = 6) by measurement of roughness average (R_a_), maximum peak-to-valley height (R_z_), and average groove depth (h) using a laser scanning microscope (VK-X110, Keyence, Osaka, Japan). Roughness parameters were analyzed by performing approximately 4 million point measurements over an area of 500 × 500 µm in the center of the blocks (50 × objectives) and the resolution was of 5 nm in y-direction.

### Flexural strength

**Fig. 3:**
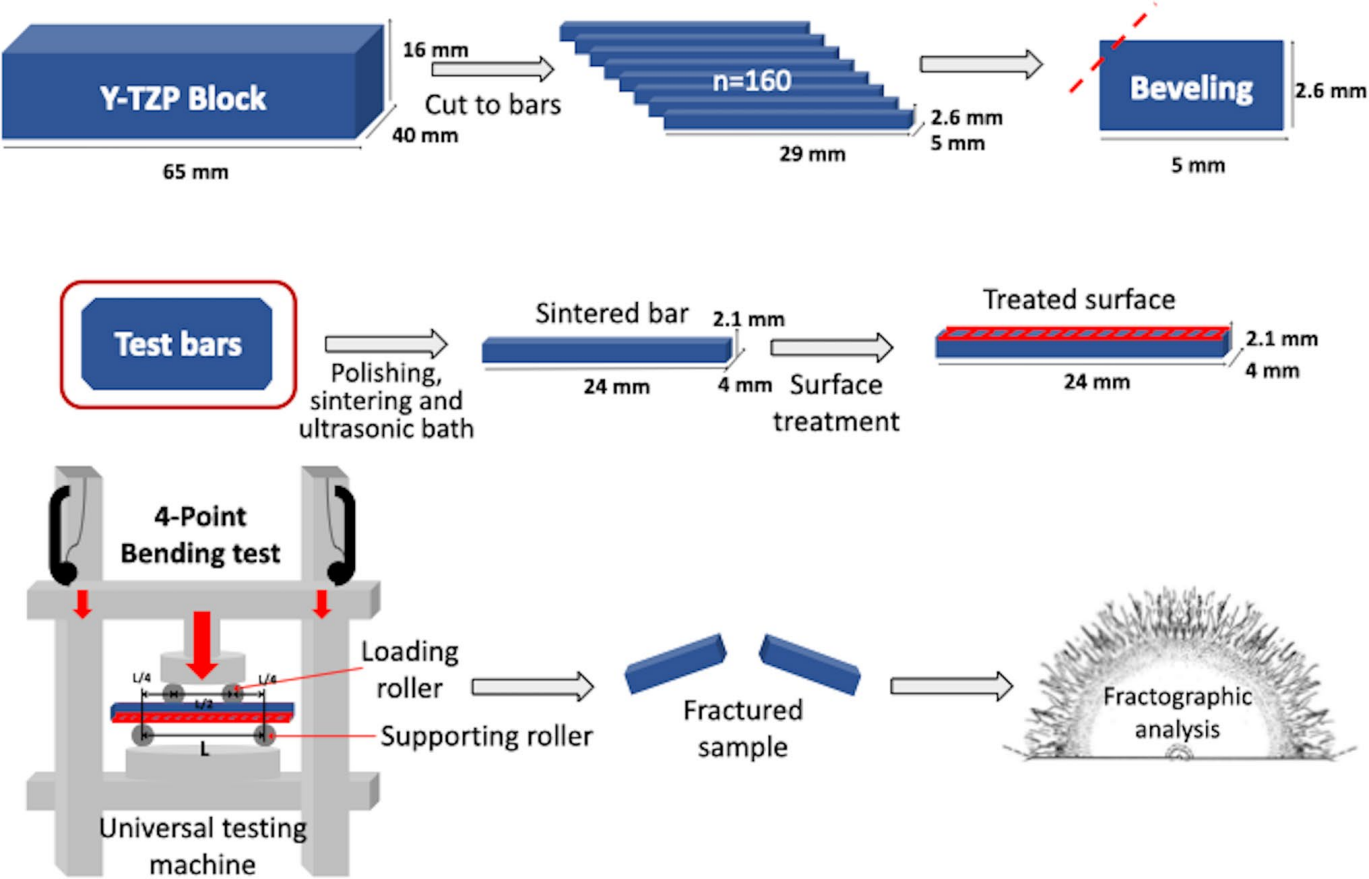
Schematic representation of the four-point flexural test according to ISO 6872. Care was taken to prepare samples with length equal 24 mm, and for beveling. Samples were placed in the universal test machine with the tested surface facing down. The distance between sample holders, L, was controlled to be = 16 mm

A four-point flexural strength test was performed according to the ISO-6872, using a universal testing machine (Instron-1186, Instron) with a 10 kN loading cell. Before the bending tests, the bars were measured again, using the same digital caliper as for sample preparation. Each sample was placed over 2 supporting rods (distance between rods: 16 mm), with the treated surface placed downward, facing the supporting device (tension side), while the untreated surface was in contact with the loading rods (compression side). The load was applied with a crosshead speed of 1 mm/min until failure, ﻿to avoid subcritical crack growth during loading, as described in the international standard [39], (Fig. 3). The maximum load (N) was recorded and the flexural strength (σ) was calculated in MPa using the following formula [39, 44]:

$${\sigma} =3{PL}/4{wb}^2,$$

where *P* is the breaking load in newtons; *L* is the center-to-center distance of the supporting rods in millimeters; *w* is the width of the sample in millimeters, and *b* is the thickness of the samples in millimeters.

### X-ray diffraction (XRD)

For crystal phase characterization, two samples per group were randomly chosen among the undestroyed samples from previous analyses. The phase identification of the sintered and treated ceramics was performed by using a X-ray diffraction device (D2-Phaser, Bruker, Billerica, Massachusetts, USA) with Cu-Kα radiation (λ = 1.56 Å) and a LynxeEye detector (PSD). The top surface of each sample was analyzed from 20 to 90° 2θ with a step size of 0.02°.﻿ The crystalline phases were qualitatively analyzed using the HighScore Plus software (Malvern PANalytical, Almelo, Netherlands) with the database PDF patterns 00–048-0224 (tetragonal lattice), 00–037-1484 (monoclinic lattice), 01–070-4436 (cubic lattice) and 00–037-1307 (rhombohedral lattice) from the international centre for diffraction data (ICDD). The amount of the tetragonal and monoclinic crystalline phases was obtained via a standard Rietveld refinement (HighScore Plus Software, Malvern Panalytical Ltd, Malvern, UK) [45], whereby also the goodness of fit *χ*^2^ could be obtained from the ratio between the weighted profile R-factor and the statistically expected *R*-value.. Once a suitable set of refinement parameters was found only the scale factor of both crystalline phases was refined for each sample to keep the results consistent. As no internal or external standard was used only the relative amounts of the crystalline phases could be determined, neglecting any contribution from the amorphous rhombohedral and cubic phases. The data plotting of the evaluated data was performed by using the software from Python Software Foundation.

### Fractured surfaces’ characterization

In order to find out the origins of zirconia bar failure, three samples from each group were randomly selected for a qualitative fractographic analysis. Their fractured surfaces were cleaned in water for 5 min using ultrasonic immersion followed by 3 min of immersion in 96% isopropanol. Subsequently, they were gold-coated for the SEM analysis at 15–20 kV, under 5–10 mbar and working distance of 14–20 mm (Leo 1455 VP, Zeiss, Oberkochen, Germany). The analyses of microscopic, fractured surface features, like fracture origin, direction of crack propagation, compression curl, and hackle lines were performed according to the current standards and the guidelines from the American Association of Dental Materials and the National Institute of Standards and Technolgy [9, 46].

### Statistical analysis

Normal distribution of data was tested using the Kolmogorov–Smirnov test (*α* = 0.05). All surface profile data showed normal distribution and were subsequently analyzed using a one-way analysis of variance (ANOVA) and a post hoc Tukey’s test (*α* = 0.05). Flexural strength data showed a non-normal distribution (Kolmogorov–Smirnov); after applying Grubbs’ outlier test, data were analyzed through both a Weibull statistical analysis [9] and Kruskall-Wallis, with Bonferroni correction for multiple tests. Weibull modulus (m) and the characteristic strength (σc) with a confidence interval of 95% were obtained through this analysis, as determined by DIN ENV 843–5 (DIN-ENV:843–5/2007). The characteristic strength is the strength at a failure probability of approximately 63%, and the Weibull modulus is used as a measure of the distribution of strengths, expressing the material's reliability [9, 47]. The statistical software SPSS Statistics 25.00 (IBM) was used for the analyses.

## Results

### Determination of ablation threshold and ablation efficiency

**Fig. 4:**
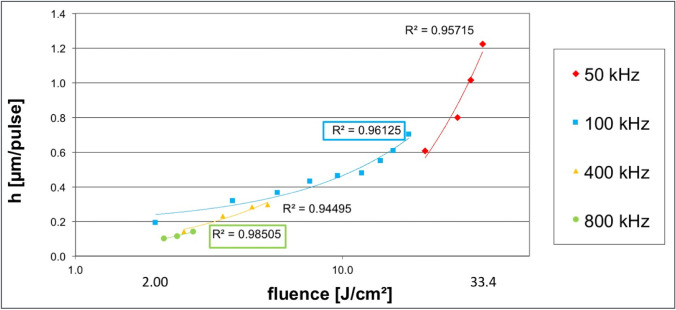
Ablation rate dependent on fluence and pulse rate. The highest ablation rate was found for 50 kHz pulse rate and the highest correlation between fluence and ablation depth was found for 100 and 800 kHz. Data fitting was done by polynomial regression

The ablation threshold data are shown in Fig. 4. The first measurable ablation was detected for fluences of around 2 J/cm^2^. For all analyzed pulse rates, there was a strong correlation between fluence and ablation depth (*R*^2 ^>0,94) after a polynomial regression fitting of the data. However, the strongest correlation was found for 800 kHz (*R*^2^ = 0.98) and 100 kHz (*R*^2^ = 0.96). The most efficient parameters found without causing surface damage (visible cracks or craters) were 2–3 J/cm^2^ for 800 kHz and 9–10 J/cm^2^ for 100 kHz.

### SEM analysis

**Fig. 5:**
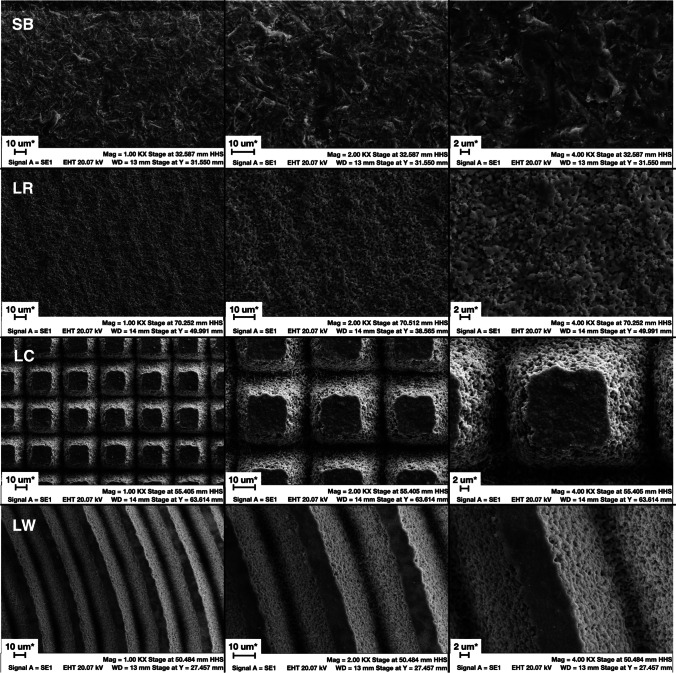
Representative SEM images of the treatment groups. Left, middle and right column with respectively 1000 × , 2000 × , and 4000 × magnification. Laser groups exhibited a very defined and homogeneous surface patterns, with very high geometrical precision and highly predictable outcomes. Even with 4000 × magnification, none of the groups exhibited mechanical flaws

USPL laser conditioning created a very homogeneous pattern throughout the zirconia surfaces. As regards morphological changes observed under SEM, laser-treated groups (crossed-lines (LC), random-hatching (LR) and parallel waves (LW)) exhibited a much more homogeneous pattern than sandblasting (SB). None of the groups showed mechanical flaws or cracks (Fig. 5).

### Surface roughness

**Fig. 6:**
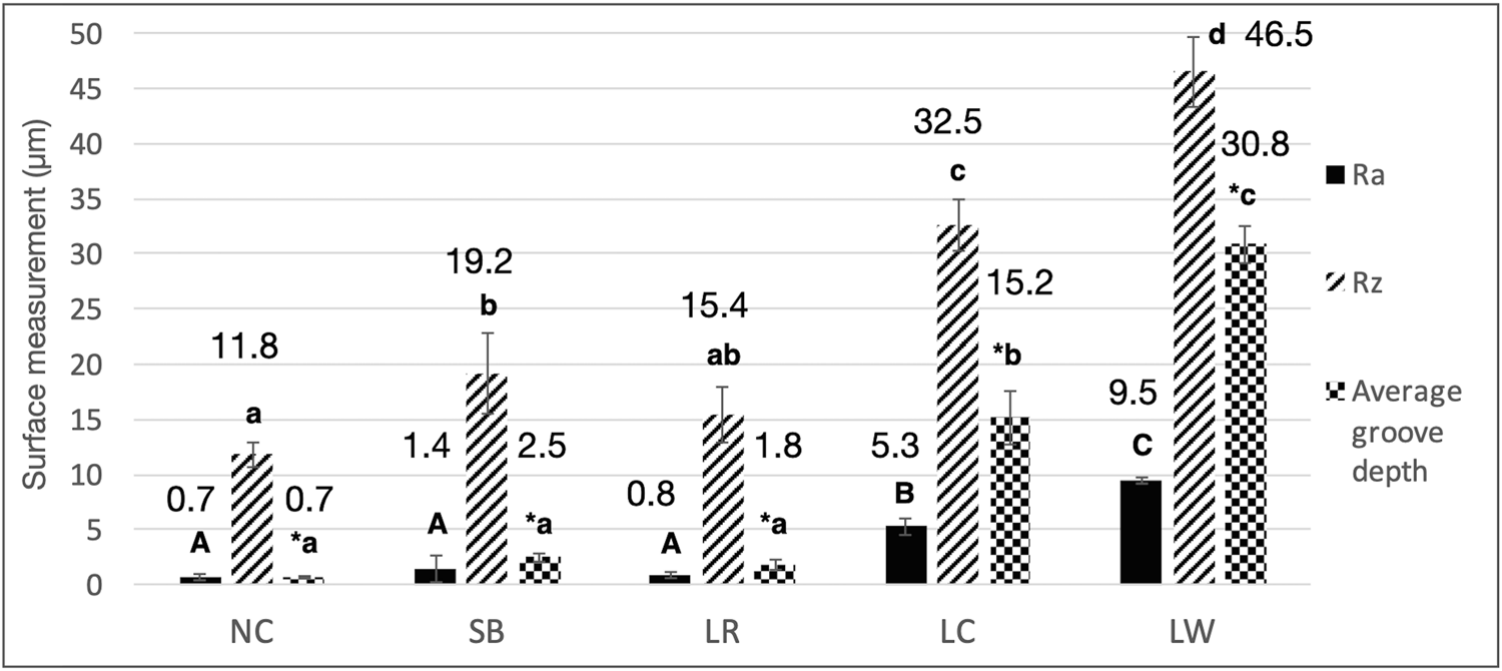
Surface roughness analysis. Means and standard deviations of Ra, Rz and groove depths (μm). In general, the lowest roughness means were observed for NC and the highest for LW. Only one laser group (LR) presented Ra, Rz and groove depth means not significantly different to both NC and SB (*p* > 0.05). Different letters indicate statistically significant differences between the groups

The mean roughness data and the groove depth are reported in Fig. 6. In general, the lowest values of Ra, Rz, and “h” were observed for NC, SB, and LR, which behaved similarly. Statistically significant increase in R_a_ was only observed for groups LC (5.3 ± 0.8 µm) and LW (9.5 ± 0.3 µm) (*p* < 0.05), whereas SB (1.4 ± 1.2 µm) and LR (0.8 ± 0.3 µm) did not significantly differ from NC (0.7 ± 0.3 µm). LR group was the only laser group that showed values of all topography parameters analyzed similar to NC (*R*_a_ p = 0.997, *R*_z_ p = 0.186, “h” *p* = 0.542) and to SB (R_a_: p = 0.616, R_z_: p = 0.136, “h”: *p* = 0.9).

### Flexural strength

**Fig. 7:**
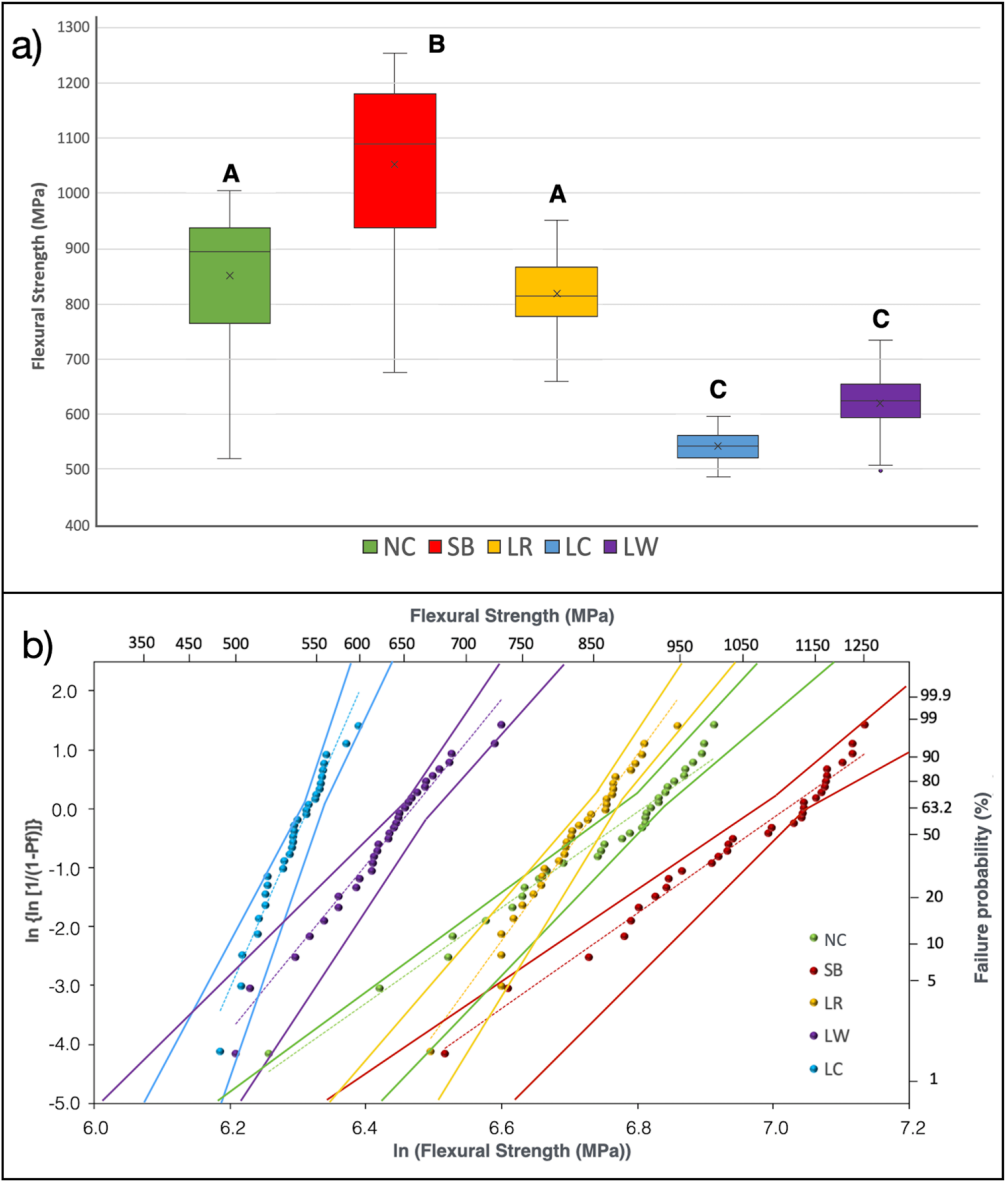
**a** Box-plot showing the median flexural strength (MPa) of 3Y-TZP after the surface treatments. Different letters indicate statistically significant differences between groups The highest flexural strength values directly after treatment were observed for SB, while LR was the only laser group showing flexural strength mean not statistically significant different to control (NC), which is known to be less susceptible to long term degradation [21, 48]. Different letters indicate statistically significant differences between the groups. **b)** Weibull plot for the four-point flexural strength (dotted lines) and the 95% confidence (straight lines). Both NC and the sandblasted group (SB) groups had the least reliability (m), while all laser groups LR, LR and LW showed higher reliability (m) than NC and SB. The highest reliability (m), indicating a narrower distribution of flexural strength data, was observed for crossed-pattern LC. LR showed the most balanced combination of reliability (*m* = 14.7) and relatively high flexural strength (Table 2)

The median flexural strength values of all groups are shown in Fig. 7a. In summary, SB showed higher flexural strength values than NC (*p* = 0.043), while LW (*p* < 0.001) and LC (*p* < 0.001) the lower flexural strength values. Only one laser group (LR) showed flexural strength means not significantly different from the non-treated NC group (*p* > 0.05).

To illustrate the difference in behavior between the groups, the Weibull distribution can be seen in graph of Fig. 7b**.** This graph shows the data dispersion (angular coefficient) as well as the coefficients of linear regression equation that were used to calculate the Weibull parameters shown in Table 2. Survival probability analysis showing that under low loads (≤ 600 MPa), LW and LC had the highest probability of failure. Under higher loads (above 900 MPa) group SB had the lowest probability of failure, while LR the most similar to the control group NC.

**Table 2:** Characteristic strength (σ_c,_ MPa), Weibull modulus (m), median four-point flexural strength (MPa), and 95% confidence intervals (95% CI) of all treated 3Y-TZP groups. Weibull distribution is shown Fig. 7b

	Characteristic Strength σ_c_^**^	95% CI of σ_c_	Weibull moduls (m)^*^	95% CI of “m*”*	Mean Flexural Strength	95% CI of Mean Flexural Strength
NC	899.5	866.9–933.3	9.8	7.3–13.1	852.0 A	809.2–894.7
SB	1113.8	1070.6–1158.8	9.2	6.9–12.3	1052.5 B	998.4–1106.6
LR	848.1	826.9–869.8	14.7	11.3–19.2	819.8 A	796.6–842.9
LC	533.3	544.6–562.0	23.7	18.3–30.8	541.6 C	532.4–550.8
LW	644.7	626.4–663.5	12.7	9.9–16.4	620.9 C	601.6–640.2

^*^The higher the m (shape value), the more consistent is the material (uniform “defects” are evenly distributed throughout the entire volume) and also the narrower the probability curve of the strength distribution [50]. ^**^The higher the characteristic strength (scale value), the highest the flexural (bending) effectiveness [50].Different letters indicate statistically significant differences

Additionally, the Weibull analysis showed (Table 2) that, as opposite to NC and SB, laser groups LC showed the highest reliability (m), but the lowest actual flexural strength. The most balanced results of having a relatively high flexural strength and reliability were observed for LR.

### Fractured surfaces characterization

**Fig. 8:**
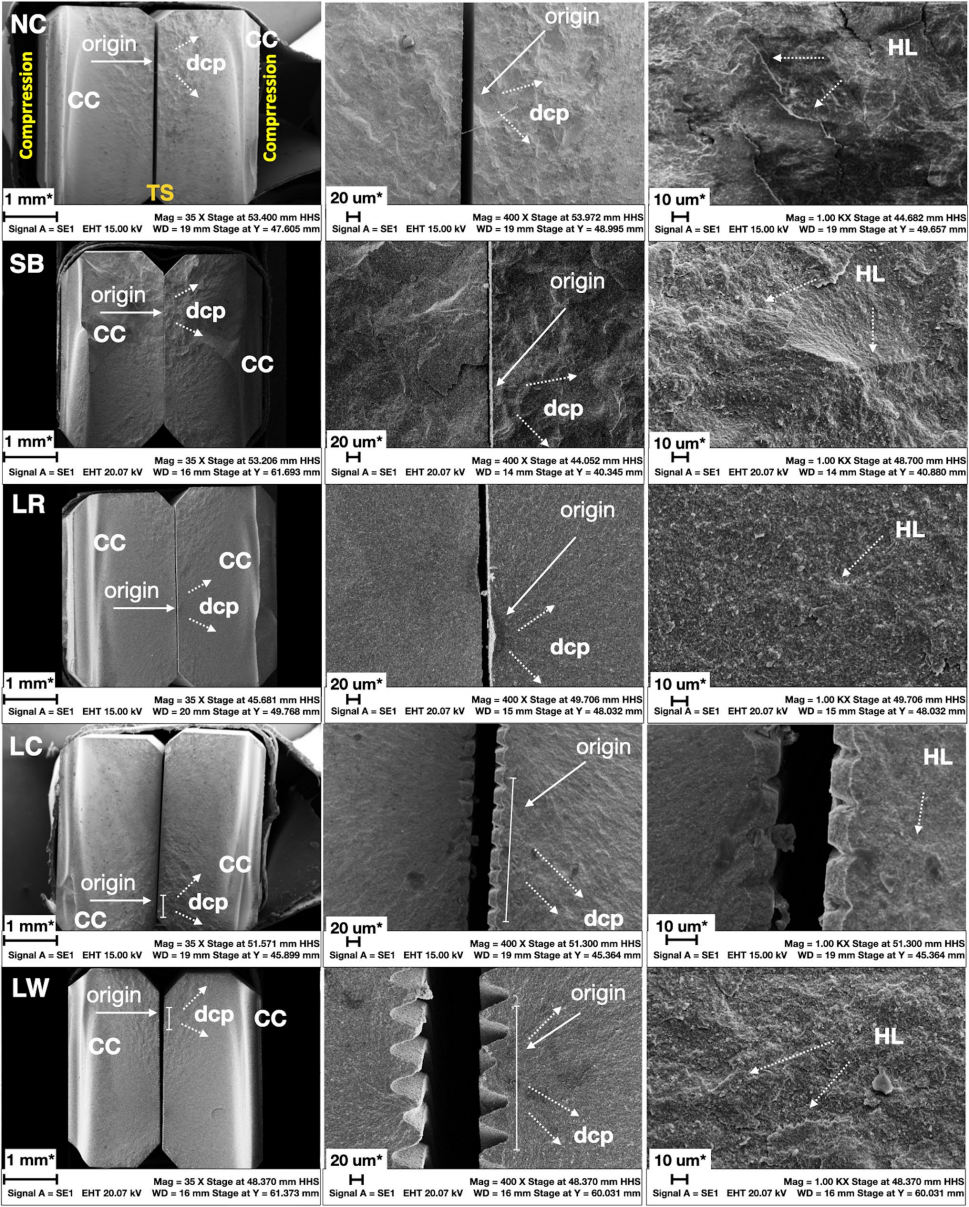
Representative SEM images of fractured 3Y-TZP bend bars. Left Column: SEM images (35 × magnification) of both the compression and the tension side (TS) of the broken bend bars. Compression curls (CC) can be seen in all groups at the opposite side of failure origin, meaning at the tension side (TS), which was also the side receiving the surface treatments (sandblasting/laser processing). Middle Column: images at intermediate magnification (400 ×) showing the fracture origin (FO) and the direction of crack propagation (dcp); for the laser patterns LC and LW the fracture origin seems to be more like a small region than a singular critical flaw. Right Column: images at higher magnification (1000 X) showing the hackle lines (HL). In the NC, SB and LR images the fracture origin at the tension side can be clearly identified, whereas in LC and LW, only the grooves generated by the laser processing are easily identifiable, but a clear singular fracture origin (critical flaw) is hard to find. In these two groups the fracture appears to originate from the tip of a group of grooves created by the laser scanning patterns. Description of fracture features according to the current guideline [46]

For all groups, failures initiated on the surface of each specimen, in the region subjected to tensile stresses during the four-point flexural strength test (Fig. 8). Compression curls (CC) opposite of failure origin, hackle lines (HL) indicating the direction of crack propagation (dcp) can be seen by all groups. However, while by NC, SB, and LR groups, the hackle lines (HL) appear to be distributed in the shape of a fan (fracture mirror) around the fracture origin (Origin), groups LC and LW showed a more parallel distribution of the hackle lines, towards the surface grooves, formed by the laser irradiation.

### X-ray diffraction (phase characterization)

**Fig. 9:**
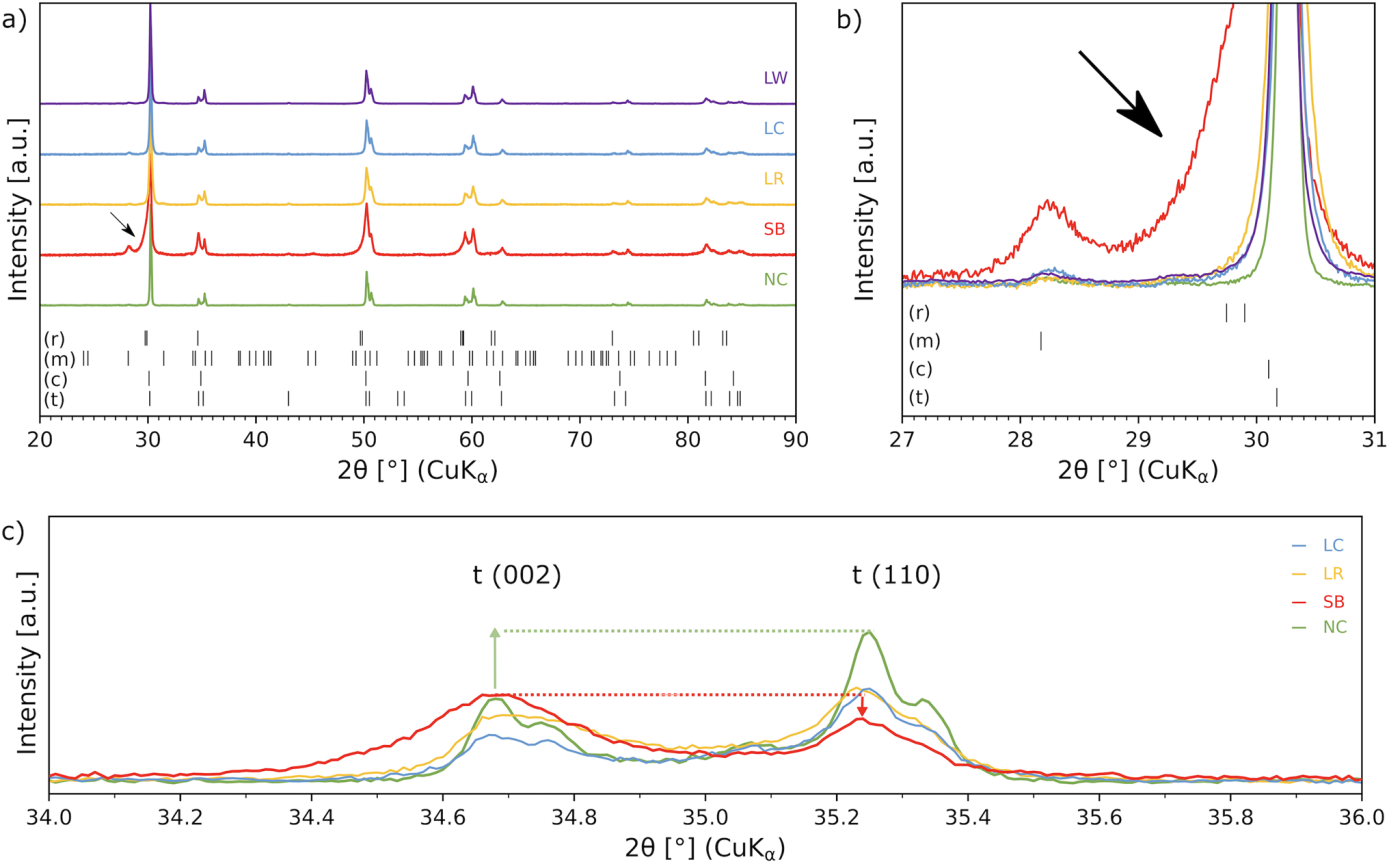
Representative XRD patterns of NC, SB, LC, LR and LW zirconia groups. **a)** SB showed clear m-ZrO2 peaks and smaller t-ZrO2 peaks, whereas NC showed the highest intensity peak for t-ZrO2 peak (30.17 degrees). **b)** All laser treated groups showed similar XRD patterns and in none of them an increase of monoclinic phase could be detected. In addition, SB showed a broadened tetragonal peak (arrow). **c)** Tetragonal peak intensity inversion after SB

The XRD patterns of each group are presented in Fig. 9a and the relative monoclinic phase contents of the treated 3Y-TZP in Table 3. Diffraction peaks for the tetragonal phase were identified in all groups. The XRD patterns of untreated 3Y-TZP ceramic surfaces (NC group) showed in the range between 2*Θ* = 30.17° and 84.84° several peaks of the tetragronal phase, with the highest peak intensity at 30.17°. A monoclinic peak at 2*Θ* = 28.17° was observed for sandblasted specimens, revealing a simultaneous decrease in the t-phase peak intensity and an asymmetrical broadening (Fig. 9b), which usually indicates the appearance of a rhombohedral phase [45]. Additionally all laser groups showed a lower monoclinic peak intensity at 28.18° than SB. Noteworthy is also a reversed intensity of the tetragonal (002) and (110) peaks observed for SB, when compared to NC or the laser groups (Fig. 9c). The treated groups, however, showed a decrease in the main intensity of the t-phase peak (30.17 degrees) compared to NC group. The highest amount of monoclinic phase was observed for sandblasting (7.2 wt.%) and the lowest for laser irradiation with the random pattern, LR (0.9 wt.%, Table 3). Both LC and LW showed a monoclinic phase content only slightly increased as compared to NC (2.1%) and considerably lower than SB, whereby the goodness of fit value was lower than 3 for all investigated groups.

**Table 3:** The relative monoclinic and tetragonal phase contents of all treated 3Y-TZP groups and the goodness of the fit of the Rietveld refinement (*χ*^2^)

	Monoclinic fraction (wt.%)	Tetragonal fraction (wt.%)	*χ*^2^
NC	2.1	97.9	2.12
SB	7.2	92.8	2.32
LR	0.9	99.1	1.82
LC	3.9	96.1	1.63
LW	4.5	95.5	2.96

## Discussion

Surface roughness measurements (R_a_, R_z_ and average groove depth) revealed that the laser groups significantly differed from non-treated control (NC), except for LR. For the flexural strength, all treatment groups resulted in values significantly different from the negative control, again, except for LR. Therefore, the null hypotheses were rejected.

For measuring the strength of ceramics, flexural tests present several advantages in comparison with tension tests. Among them, the lower cost of specimen fabrication and lesser difficulty in gripping without introducing bending and damaging contact stresses [48]. In this study, a four-point flexural test was used. Since the volume under stress for the four-point bending test is larger than for the three-point test and the stress applied is uniformly distributed over the whole specimen [49], there is a higher probability to find a longer crack or flaw, according to Weibull statistics, which makes it a more reliable test for ceramic materials, which are very brittle and very difficult to prepare for pure tensile tests [47].

Although USPL laser conditioning could be an alternative method for roughening the zirconia surface and favoring adhesion of composite cements [26, 27] as well as improving osteointegration of dental implants [28, 29], it was up to now unclear if certain laser-created microsturctures could be disadvantageous for the flexural strength of the irradiated material. Also, the amount of transformation of tetragonal to monoclinic transformation at the zirconia surface has not been investigated up to now in dependence of the type of surface patterns created through laser microstructuring. Both the influence of picosecond laser-created surface patterns on flexural strength and *t-m* transformation of 3Y-TZP ceramic were tested to the best of our knowledge for the first time in the present study for these newly described laser irradiation settings. Indeed, after the four-point flexural test, one of the laser patterns tested, namely random hatching (LR), not only achieved mean flexural strength above the minimum accepted for clinical use in multiple elements restorations (710 MPa), as reported in the international standard (ISO-6872/2015) [39] but also, did not significantly differ from the as-sintered ceramic (NC). Furthermore, the Weibull modulus (m) of LR increased, while the amount of monoclinic content decreased as compared to NC. The latter might indicate that the fabrication defects (introduced during machining partly sintered zirconia blocks) and the compressive stress zone were partly removed, since this group was the only that completely removed a very thin layer of the surface. Regarding surface morphology, LR was also the most similar to SB among the laser groups, which could be an advantage in terms of increasing bond strength to composite cements; however, this aspect is yet unknown.

In general, all laser groups exhibited higher Weibull moduli than NC and SB, although LC and LW showed lower flexural strength values. The higher the “m” (shape value), the more consistent is the material, meaning that the “defects” are more uniformly distributed throughout the material [50]. Thus, the increased “m” values of all laser groups actually reflect the capacity of the laser automated processing in generating very defined and homogeneous surface patterns, with very high geometrical precision and highly predictable influence on flexural strength. Among the laser groups LC had the highest m value, and this is in accordance with the kind of surface pattern observed (Fig. 2), as exact symmetric crossing parallel lines in both horizontal and vertical direction were created. As in the present study, this was the pattern with most symmetrical and homogeneous distribution of the surface microstructures in both directions; the increased *m* value seems to be clearly correlated with this geometrical distribution. Additionally, the fact that SB showed significantly higher flexural strength means than NC is also in accordance with other studies and validates the current four-point-flexural strength model [13, 15–17, 51].

It is important to note that also some limitations are related to this laser-microstructuring approach. For example, there are currently only initial concepts about how the USPL lasers could be included in the clinical flow of preparing and inserting indirect restorations. In the present study, flat zirconia surfaces were laser irradiated, which is much simpler than laser conditioning the internal walls of an indirect restoration. However, this issue is already well-developed and achieved with very high precision for zirconia implants, due to the development of a tailor-made quasi-tangential process for the laser microstructuring of dental implants with varying geometries [25]. Therefore, there are reasons to believe, that is will be also more easily achievable with zirconia restorations.

Many factors influence the strength of zirconia ceramics and at some level it is probably determined by the balance between microcrack formation (decreased strength) and surface compressive stress build-up (increased strength) [52]. However, one can also observe how surface roughness influences zirconia’s strength, as it can be related both microcracks formation as well as t-m transformation and consequently surface compressive stress. When this level of correlation is considered, an increase in surface roughness has been often related to a decrease in flexural strength, meaning that an inverse correlation between roughness and flexural strength has been frequently observed [12, 14–16]. Such an inverse correlation between flexural strength and roughness was also observed in the present study (*r*^2^ = from 0.62 to 0.9) for all laser groups. This phenomenon has been explained by Quinn [9], who stated that when the introduced cracks are deeper than the existing surface flaws, a stronger correlation between roughness and flexural strength is noticed. This may explain why there is a stronger correlation for group LC, for example (*r*^2^ = 0.88), that presented distinct linear grooves created by laser ablation, than for group LR (*r*^2^ = 0.62), which exhibited only a roughened surface, with no visible grooves. Considering this, it is reasonable to believe, that further reducing the Rz value of the laser groups could further increase its flexural strength and new studies testing this hypothesis should be conducted.

XRD analysis of SB showed a broadened tetragonal peak, implying the presence of rhombohedral or a distorted tetragonal zirconia on the surface [45]. This broadened t-ZrO_2_ peak in 3 mol% Y-TZP was reported in several studies particularly after surface treatments, such as sandblasting [45, 53]. Regarding the m-phase, only SB showed considerably increased monoclinic peaks. The tetragonal-to-monoclinic phase (*t-m*) transformation that occurred, with a stronger m-phase peak for SB, and its reduced intensity for the non-treated and laser-treated groups, may explain the significantly higher flexural strength observed for SB in the present study [3, 5, 13]. On the other hand, the preservation of the m-phase content after laser irradiation of 3Y-TZP ceramic is also in accordance with other studies, in which similar or lower m-phase content has been observed after nano- [35] as well after picosecond laser microstructuring [24] using other surface patterns. Especially interesting is the fact, that one of the laser patterns, LR, even slightly reduced the m-phase content of the zirconia surface. This might be specially interesting in a clinical situation, as it consequently means that there will be a higher tetragonal phase content at the surface of the dental zirconia, which could be transformed to monoclinic in the presence mechanical load (as for example chewing stress), causing volume expansion and stopping crack propagation, thus increasing strength, when it is clinically needed.

The occurrence of *t-m* transformation previous to clinical use, as it occurs after sandblasting, is not always advantageous for the material. According to the literature, when the sandblasting treatment generates monoclinic content lower than 12 to 25%, the long-term flexural strength is not affected, because tetragonal to monoclinic transformation occurred only on the outer surface [21]. Although this transformation should be primarily favorable to the material’s mechanical properties, in a higher percentage, it can impair strength of 3Y-TZP structures, since the t-m transformation can spread throughout the material, resulting in grain pullout and higher roughness [15]. The transformation of each grain is accompanied by volume increase, causing stresses on the surrounding grains and microcracking. With water penetration, the process of degradation and the transformation progresses from particle to particle can be intensified and has been called low-temperature degradation [22]. Furthermore, since two different crystal structures occur after the *t-m* phase transformation, the coefficients of thermal expansion of the different phases show discrepancies, which also affects the bonding capability of the zirconia [15, 51]. This could mean that, in the long term, the greater t-m phase transformation that occurred in the present study for SB could be prejudicial to both flexural and bonding strength of the material. However, this is still controversial and other experiments have shown that only Y-TYP/Alumina zirconia and not 3Y-TZP was more susceptible to aging after sandblasting, thus future studies are still need to clarify this [20].

As opposite to that, group LR showed flexural strength above the minimum accepted for clinical use in multiple elements [39, 54], without increasing the content of m-phase and, therefore, it is reasonable to speculate that it may offer the most stable flexural resistance in the long run. If this is indeed correct, must be still investigated in laboratorial (in vitro) and pre-clinical studies. It is interesting to observe though that this assumption is in accordance with the findings of a recent study [51], which showed that the initial higher flexural strength of air-abraded zirconia decreased (strength degradation) with increasing mechanical load (simulating clinical aging), while the flexural strength of non-treated control remained constant. In this way both treatments resulted over time in flexural strength means that were in a remarkably similar level [51].

In summary, the newly described laser processing strategy, random-hatching (LR), enables extremely short conditioning-times of the 3Y-TZP ceramic (only 5 s/ repetition), due to the extreme short pulses (only some trillionths of a second), very high frequencies (hundreds of kilohertz) and very high scan speed (thousands of mm/s). Furthermore, the ultra-short pulsed laser microstructuring under LR conditions created very predictable, homogeneous, highly reproducible and accurate surface micro structures on dental zirconia ceramic. This automated laser microstructuring strategy (random-hatching, 12 ps pulses) increased 3Y-TZP average surface roughness similarly to SB, while not causing deleterious crystal phase transformation or loss of flexural strength and increasing the Weibull modulus and consequently material’s reliability.

## References

[1] Denry I, Kelly JR (2008). State of the art of zirconia for dental applications. Dent Mater.

[2] Zhang Y, Lawn BR (2018). Novel zirconia materials in dentistry. J Dent Res.

[3] Chevalier J, Gremillard L, Virkar AV, Clarke DR (2009). The tetragonal-monoclinic transformation in zirconia: lessons learned and future trends. J Am Ceram Soc.

[4] Zhang F, Van Meerbeek B, Vleugels J (2020). Importance of tetragonal phase in high-translucent partially stabilized zirconia for dental restorations. Dent Mater.

[5] Hannink RHJ, Kelly PM, Muddle BC (2000). Transformation toughening in zirconia-containing ceramics. J Am Ceram Soc.

[6] Belli R, Loher C, Petschelt A, Cicconi MR, de Ligny D, Anglada M, Lohbauer U (2020). Low-temperature degradation increases the cyclic fatigue resistance of 3Y-TZP in bending. Dent Mater.

[7] Garvie RC, Hannink RH, Pascoe RT (1975). Ceramic steel. Nature.

[8] Lohbauer U, Amberger G, Quinn GD, Scherrer SS (2010). Fractographic analysis of a dental zirconia framework: a case study on design issues. J Mech Behav Biomed.

[9] Quinn G (2016) A NIST recommended practice guide:fractography of ceramics and glasses. Special publication960–16e2. Washington, DC: National Institute of Standardsand Technology; http://nvlpubs.nist.gov/

[10] Inokoshi M, De Munck J, Minakuchi S, Van Meerbeek B (2014). Meta-analysis of bonding effectiveness to zirconia ceramics. J Dent Res.

[11] Kern M (2015). Bonding to oxide ceramics-laboratory testing versus clinical outcome. Dent Mater.

[12] Wang Y, Lam WYH, Luk HWK, Oilo M, Shih K, Botelho MG (2020). The adverse effects of tungsten carbide grinding on the strength of dental zirconia. Dent Mater.

[13] Kosmac T, Oblak C, Jevnikar P, Funduk N, Marion L (1999). The effect of surface grinding and sandblasting on flexural strength and reliability of Y-TZP zirconia ceramic. Dent Mater.

[14] Botelho MG, Dangay S, Shih K, Lam WYH (2018). The effect of surface treatments on dental zirconia: An analysis of biaxial flexural strength, surface roughness and phase transformation. J Dent.

[15] Okada M, Taketa H, Torii Y, Irie M, Matsumoto T (2019). Optimal sandblasting conditions for conventional-type yttria-stabilized tetragonal zirconia polycrystals. Dent Mater.

[16] Aurelio IL, Marchionatti AM, Montagner AF, May LG, Soares FZ (2016). Does air particle abrasion affect the flexural strength and phase transformation of Y-TZP? A systematic review and meta-analysis. Dent Mater.

[17] Cotic J, Jevnikar P, Kocjan A, Kosmac T (2016). Complexity of the relationships between the sintering-temperature-dependent grain size, airborne-particle abrasion, ageing and strength of 3Y-TZP ceramics. Dent Mater.

[18] Denry I (2013). How and when does fabrication damage adversely affect the clinical performance of ceramic restorations?. Dent Mater.

[19] Deville S, Chevalier J, Gremillard L (2006). Influence of surface finish and residual stresses on the ageing sensitivity of biomedical grade zirconia. Biomaterials.

[20] Inokoshi M, Vanmeensel K, Zhang F, De Munck J, Eliades G, Minakuchi S, Naert I, Van Meerbeek B, Vleugels J (2015). Aging resistance of surface-treated dental zirconia. Dent Mater.

[21] Sawada T, Schille C, Zoldfoldi J, Schweizer E, Geis-Gerstorfer J, Spintzyk S (2018). Influence of a surface conditioner to pre-sintered zirconia on the biaxial flexural strength and phase transformation. Dent Mater.

[22] Chevalier J, Gremillard L, Deville S (2007). Low-temperature degradation of Zirconia and implications for biomedical implants. Annu Rev Mater Res.

[23] Zhou HB, Li C, Zhou ZK, Cao RY, Chen Y, Zhang SS, Wang G, Xiao S, Li Z, Xiao P (2018). Femtosecond laser-induced periodic surface microstructure on dental zirconia ceramic. Mater Lett.

[24] Delgado-Ruiz RA, Calvo-Guirado JL, Moreno P, Guardia J, Gomez-Moreno G, Mate-Sanchez JE, Ramirez-Fernandez P, Chiva F (2011). Femtosecond laser microstructuring of zirconia dental implants. J Biomed Mater Res B Appl Biomater.

[25] Ackerl N, Warhanek M, Gysel J, Wegener K (2019). Ultrashort-pulsed laser machining of dental ceramic implants. J Eur Ceram Soc.

[26] Abu Ruja M, De Souza GM, Finer Y (2019). Ultrashort-pulse laser as a surface treatment for bonding between zirconia and resin cement. Dent Mater.

[27] Esteves-Oliveira M, Jansen P, Wehner M, Dohrn A, Bello-Silva MS, Eduardo CP, Meyer-Lueckel H (2016). Surface Characterization and Short-term Adhesion to Zirconia after Ultra-short Pulsed Laser Irradiation. J Adhes Dent.

[28] Delgado-Ruiz RA, Gomez Moreno G, Aguilar-Salvatierra A, Markovic A, Mate-Sanchez JE, Calvo-Guirado JL (2016). Human fetal osteoblast behavior on zirconia dental implants and zirconia disks with microstructured surfaces. An experimental in vitro study. Clin Oral Implants Res.

[29] Calvo-Guirado JL, Aguilar-Salvatierra A, Delgado-Ruiz RA, Negri B, Fernandez MP, Sanchez M, de Val JE, Gomez-Moreno G, Romanos GE (2015). Histological and histomorphometric evaluation of zirconia dental implants modified by femtosecond laser versus titanium implants: an experimental study in fox hound dogs. Clin Implant Dent Relat Res.

[30] Parry JP, Shephard JD, Hand DP, Moorhouse C, Jones N, Weston N (2011). Laser micromachining of zirconia (Y-TZP) ceramics in the picosecond regime and the impact on material strength. Int J Appl Ceram Tec.

[31] Barsch N, Werelius K, Barcikowski S, Liebana F, Stute U, Ostendorf A (2007). Femtosecond laser microstructuring of hot-isostatically pressed zirconia ceramic. J Laser Appl.

[32] Werelius K, Weigl P, Lubatschowski H (2003) Processing HIP-zirconia with ultra-short laser pulses. Fourth international symposium on laser precision microfabrication. Proceedings of the SPIE 5063:342–345. 10.1117/12.540505

[33] Ji M, Xu JY, Chen M, El Mansori M (2020). Enhanced hydrophilicity and tribological behavior of dental zirconia ceramics based on picosecond laser surface texturing. Ceram Int.

[34] Roitero E, Lasserre F, Anglada M, Mucklich F, Jimenez-Pique E (2017). A parametric study of laser interference surface patterning of dental zirconia: effects of laser parameters on topography and surface quality. Dent Mater.

[35] Pereira RSF, Moura CG, Henriques B, Chevalier J, Silva FS, Fredel MC (2020). Influence of laser texturing on surface features, mechanical properties and low-temperature degradation behavior of 3Y-TZP. Ceram Int.

[36] Knappe V, Frank F, Rohde E (2004). Principles of lasers and biophotonic effects. Photomed Laser Surg.

[37] Kramer T, Remund S, Jaggi B, Schmid M, Neuenschwander B (2018). Ablation dynamics - from absorption to heat accumulation/ultra-fast laser matter interaction. Adv Opt Technol.

[38] Roitero E, Anglada M, Mucklich F, Jimenez-Pique E (2018). Mechanical reliability of dental grade zirconia after laser patterning. J Mech Behav Biomed Mater.

[39] International Organisation for Standardization [ISO-6872]: Dentistry - Ceramic Materials (2015) https://www.iso.org/standard/59936.html

[40] Lopez-Suarez C, Castillo-Oyague R, Rodriguez-Alonso V, Lynch CD, Suarez-Garcia MJ (2018). Fracture load of metal-ceramic, monolithic, and bi-layered zirconia-based posterior fixed dental prostheses after thermo-mechanical cycling. J Dent.

[41] Esteves-Oliveira M, Wollgarten S, Liebegall S, Jansen P, Bilandzic M, Meyer-Lueckel H, Fischer H, Stollenwerk J, Poprawe R (2017). A new laser-processing strategy for improving enamel erosion resistance. J Dent Res.

[42] Bello-Silva MS, Wehner M, Eduardo Cde P, Lampert F, Poprawe R, Hermans M, Esteves-Oliveira M (2013). Precise ablation of dental hard tissues with ultra-short pulsed lasers. Preliminary exploratory investigation on adequate laser parameters. Lasers Med Sci.

[43] Esteves-Oliveira M, Apel C, Gutknecht N, Velloso WF, Cotrim MEB, Eduardo CP, Zezell DM (2008). Low-fluence CO_2_ laser irradiation decreases enamel solubility. Laser Phys.

[44] Kurtulmus-Yilmaz S, Aktore H (2018). Effect of the application of surface treatments before and after sintering on the flexural strength, phase transformation and surface topography of zirconia. J Dent.

[45] Inokoshi M, Shimizu H, Nozaki K, Takagaki T, Yoshihara K, Nagaoka N, Zhang F, Vleugels J, Van Meerbeek B, Minakuchi S (2018). Crystallographic and morphological analysis of sandblasted highly translucent dental zirconia. Dent Mater.

[46] Scherrer SS, Lohbauer U, Della Bona A, Vichi A, Tholey MJ, Kelly JR, van Noort R, Cesar PF (2017). ADM guidance-Ceramics: guidance to the use of fractography in failure analysis of brittle materials. Dent Mater.

[47] Quinn JB, Quinn GD (2010). A practical and systematic review of Weibull statistics for reporting strengths of dental materials. Dent Mater.

[48] Turon-Vinas M, Anglada M (2018). Strength and fracture toughness of zirconia dental ceramics. Dent Mater.

[49] Miura D, Ishida Y, Miyasaka T, Aoki H, Shinya A (2020) Reliability of different bending test methods for dental press ceramics. Materials (Basel) 13(22):5162. 10.3390/ma13225162 10.3390/ma13225162 PMC7696483 33207710

[50] Inokoshi M, Zhang F, Vanmeensel K, De Munck J, Minakuchi S, Naert I, Vleugels J, Van Meerbeek B (2017). Residual compressive surface stress increases the bending strength of dental zirconia. Dent Mater.

[51] Amaral M, Cesar PF, Bottino MA, Lohbauer U, Valandro LF (2016). Fatigue behavior of Y-TZP ceramic after surface treatments. J Mech Behav Biomed Mater.

[52] Inokoshi M, Shimizubata M, Nozaki K, Takagaki T, Yoshihara K, Minakuchi S, Vleugels J, Van Meerbeek B, Zhang F (2021). Impact of sandblasting on the flexural strength of highly translucent zirconia. J Mech Behav Biomed Mater.

[53] Cotic J, Jevnikar P, Kocjan A (2017). Ageing kinetics and strength of airborne-particle abraded 3Y-TZP ceramics. Dent Mater.

[54] Zarone F, Di Mauro MI, Spagnuolo G, Gherlone E, Sorrentino R (2020) Fourteen-year evaluation of posterior zirconia-based three-unit fixed dental prostheses: A prospective clinical study of all ceramic prosthesis. J Dent 101:103419. 10.1016/j.jdent.2020.103419 10.1016/j.jdent.2020.103419 32619571

